# Controlling the Er content of porous silicon using the doping current intensity

**DOI:** 10.1186/1556-276X-9-332

**Published:** 2014-07-04

**Authors:** Guido Mula, Lucy Loddo, Elisa Pinna, Maria V Tiddia, Michele Mascia, Simonetta Palmas, Roberta Ruffilli, Andrea Falqui

**Affiliations:** 1 Dipartimento di Fisica, Cittadella Universitaria di Monserrato, Università degli Studi di Cagliari, S.P. 8 km 0.7, Monserrato, Cagliari 09042, Italy; 2 Dipartimento di Ingegneria Meccanica Chimica e dei Materiali, Università degli Studi di Cagliari, Piazza d’Armi, Cagliari 09123, Italy; 3 Nanochemistry, Istituto Italiano di Tecnologia, Via Morego 30, Genova 16163, Italy; 4 Biological and Environmental Sciences and Engineering Division (BESE), King Abdullah University of Science and Technology (KAUST), Thuwal, Jeddah, Kingdom of Saudi Arabia

**Keywords:** Porous Silicon, Er doping, Electrochemical impedance spectroscopy, Reflectivity, Scanning electron microscopy

## Abstract

The results of an investigation on the Er doping of porous silicon are presented. Electrochemical impedance spectroscopy, optical reflectivity, and spatially resolved energy dispersive spectroscopy (EDS) coupled to scanning electron microscopy measurements were used to investigate on the transient during the first stages of constant current Er doping. Depending on the applied current intensity, the voltage transient displays two very different behaviors, signature of two different chemical processes. The measurements show that, for equal transferred charge and identical porous silicon (PSi) layers, the applied current intensity also influences the final Er content. An interpretative model is proposed in order to describe the two distinct chemical processes. The results can be useful for a better control over the doping process.

**PACS:**

81.05.Rm; 82.45.Rr

## Background

The rare earth doping of Si as a means to obtain efficient light emission 1.5 μm has attracted a lot of interest [1-7] since, given its indirect bandgap, Si photoluminescence can be obtained only through strong quantum confinement [8]. Porous silicon (PSi) studies already reported interesting Er-related photoluminescence [2,9-11] or electroluminescence [12]. Unfortunately, this research activity did not lead, till now, to market-valuable devices, basically because almost no research has been devoted to the understanding of the doping process itself. Most studies, even very recent ones [11], use only optical properties as a means to optimize the Er doping process on bulk Si [10] or PSi [3,9]. However, given the large internal surface of the material, the electrochemical doping of PSi is a quite complex process that we are just beginning to understand: all we have are just a few studies on the cyclic voltammetry of the Er deposition process [13], on the effect of doping duration [7], and on the evolution of the doping process as a function of several parameters [14,15].

The luminescence in itself being not an issue, we focused our study on the control of the electrochemical doping process of PSi. We will show that gaining detailed information about the early stages of the process is instrumental for understanding the final results of the doping process and the key for its optimization. In our study, significant information in the understanding of these early stages is obtained by electrochemical impedance spectroscopy (EIS). This is a very valuable technique for porous materials [16-20] and has already been successfully applied to PSi for the study of cyclic oxidation [21,22].

## Methods

PSi layers were prepared by electrochemical etching in the dark of *n*^+^-doped (100)-oriented crystalline Si wafers having 3 to 7 mΩ/cm resistivity from Siltronix (Archamps, France). The etched bulk Si surface area is about 0.9 cm^2^. The etching solution was HF/H_2_O/ethanol in a 15/15/70 proportion, respectively, and the etching current density was 50 mA/cm^2^ in all cases. HF being an extremely hazardous material (e.g., see [23]), all precautions have been taken to ensure the safety of the persons involved in the porous samples preparation.

The Er doping was performed in constant current configuration with current densities in the 0.01 to 2.2 mA/cm^2^ range using a 0.11 M solution of $Er{\left(NO_{3}\right)}_{3}\;.\;5H_{2}O$ in EtOH. EIS measurements and Er doping processes were always performed with the same electrochemical cell used for the PSi formation. The Er solution used was also the same in both cases. The EIS measurements were made in the galvanostatic regime (GEIS) using a constant bias current in the 0.01 to 1 mA range, a frequency range from 100 kHz to 100 mHz, and an AC amplitude of 2 to 10 μA, depending on the bias current intensity.

All electrochemical processes were performed using a PARSTAT 2273 potentiostat by Princeton Applied Research (Oak Ridge, TN, USA). A schematic of the cell used for the experiments can be found in [14].

Spatially resolved energy dispersive spectroscopy (EDS) measurements for quantitative Er content determination were carried out using a JEOL JED 2300 Si(Li) detector in a scanning electron microscope (SEM) JEOL JSM 6490-LA (JEOL Ltd., Akishima, Japan) equipped with a W thermionic electron source and working at an acceleration voltage of 15 kV.

The fitting of the reflectivity spectra was performed using the SCOUT software from W. Theiss Hard- and Software (Aachen, Germany).

## Results and discussion

### Optical characterization

**Figure 1:**
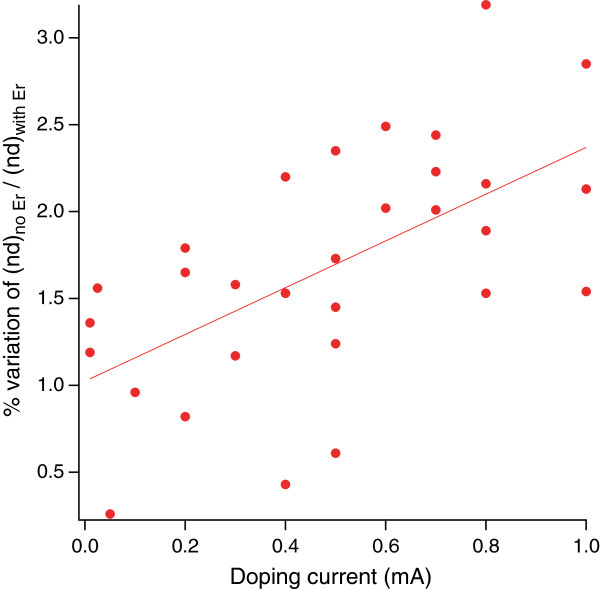
**Evolution of the PSi optical thickness nd as a function of the doping current.** The red circles are the ratio of the nd values (*n* is the refractive index and *d* the physical thickness) before and after the doping process. The transferred charge is the same for all samples. The line fit is to be intended as a guide for the eyes.

The presence of Er within the PSi pores induces a modification of the optical response of the material that is correlated to the amount of Er present in the layers [14]. To gain information about the modifications of the PSi/Er doping process as a function of the doping current intensity, we performed a series of reflectivity measurements on samples where we transferred, using different current intensities, equal amounts of charge during the electrochemical process. We have then fitted the reflectivity spectra, using the SCOUT software, to obtain the variation of the optical thickness following the Er doping. Each sample has been measured before and after the doping process, so that the results are independent on small differences in the thickness from one sample to another. The results are shown in Figure 1, where (nd)_no Er_ and (nd)_with Er_ refer to the optical thickness before and after the doping, respectively, nd being the product of the refractive index *n* and the layer thickness *d*.

If the doping process were independent on the doping current, the data should follow a horizontal line, since no evolution would be expected. However, our results, even with the large spread, indicate that there is a clear trend, although a fully quantitative determination cannot be obtained. It must be noted that a spread in the data is expected because there are several small parameters that can affect the results. For instance, the minute differences in the surface/bulk properties of the starting Si wafer will affect the shape of the pore openings and, in turn, the diffusion of the Er solution within the pores. This effect is also expected for samples coming from different parts of the starting Si wafer (32 samples are obtained for each 4-in. wafer). The line fit is shown as a guide for the eyes to evidence the trend. Given the correlation of the samples optical properties with their Er content [14,15], based on the data of Figure 1, we can get a first hint that this evolution indicates a current intensity-dependent Er content.

### Electrochemical characterization

**Figure 2:**
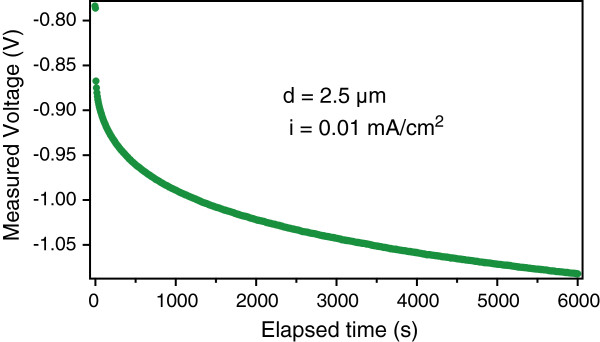
**Voltage evolution in PSi Er doping using a low constant current intensity.** The time evolution of the applied voltage indicates the presence of a single transient.

**Figure 3:**
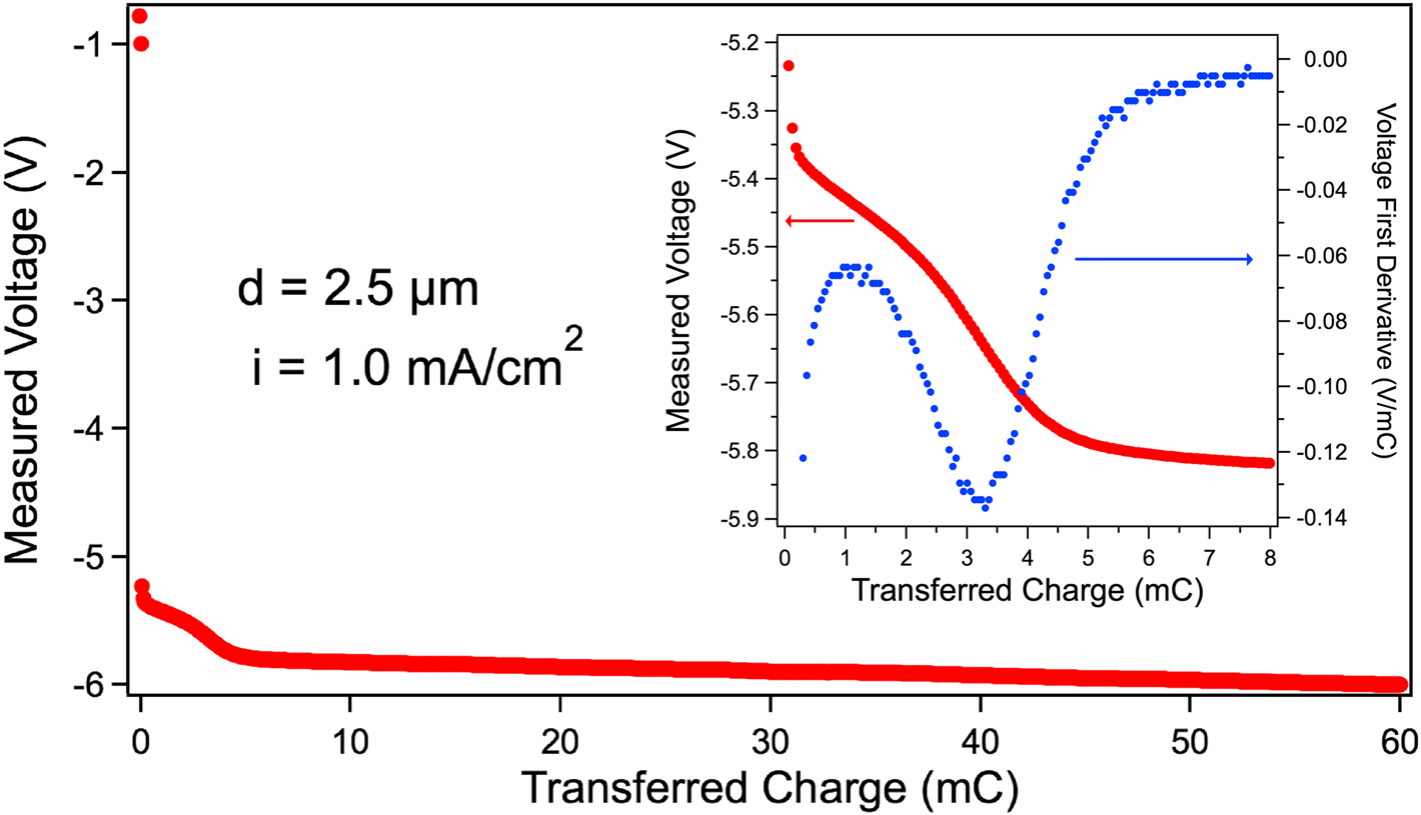
**Voltage evolution in PSi Er doping using a high constant current intensity.** The presence of a double transient is evident. In the inset, the first derivative of the curve (blue dotted line, right axis) is shown superposed to the original curve (red dotted line, left axis) to highlight the slope change induced by the presence of the double transient.

Figures 2 and 3 show the measured voltage transients for applied currents with low and high densities, respectively, in two nominally identical PSi samples (2.5-μm thick). The total transferred charge is the same for both transients. The inset of Figure 3 shows an enlargement of the plot of Figure 3 (red dots) superposed to its first derivative (blue dots). The same effect has been observed for several other thicknesses.The results of Figures 2 and 3 demonstrate the existence of two different transient shapes: at low currents, a single transitory (ST) is evidenced by the regular increase of the voltage absolute value (Figure 2), while a double transitory (DT) is evidenced for higher currents (Figure 3), where a variation in the slope during the voltage evolution is clearly visible also as a clear peak in its first derivative (inset of Figure 3). The presence for higher currents of a slope change indicates that two different Er deposition processes are involved, while a single regime is present for lower currents. Although to date the onset of the transition between the two regimes as a function of the doping parameters is not clearly definite, we observed that all higher current density doping processes exhibit a DT, while all lower current ones exhibit a ST. We also observed that the DT shape depends on the current intensity and that there is a correlation of the shape with the current density (not shown).

To gain further insight in the differences between ST and DT regimes, we studied the evolution of the first stages of the doping process by means of GEIS. GEIS spectroscopy is a very useful technique with high sensitivity to surface changes and well suitable to the characterization of porous materials: it allows analyzing the response of the samples under a wide frequency window. Moreover, the equivalent circuit approach was used to interpret the mechanism of the process. Parallel–series combinations of circuital electrical elements are used to simulate the response. Resistors (R) and capacitors (C) are mainly adopted but also constant phase element (CPE) is often used, instead of C, to take account for possible non-ideality of the capacitor behavior: their admittance is expressed by *Y* = *Q* (jω)^*n*^, the value of *n* being 1 for perfect capacitors [18].

**Figure 4:**
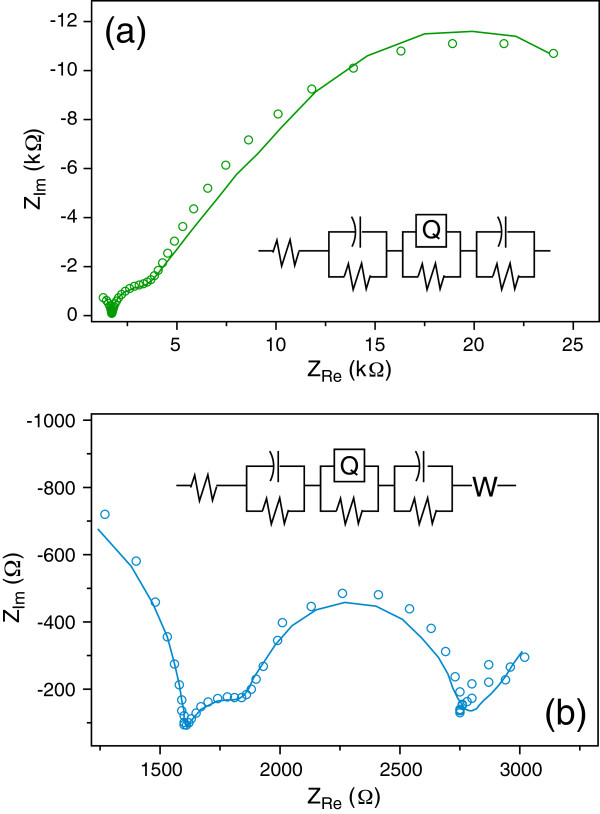
**Comparison between fitted circuit models and measured Nyquist data obtained during doping at low (a) and high (b) current intensities.** The equivalent circuit adopted is also shown as inset. Experimental data are the 4th and 3rd GEIS cycles of Figures 5a and 6b, respectively.

Figure 4a shows an example of the typical Nyquist plot obtained during a low current doping: the data are the empty circles while the full line represents the results of the fitting obtained by the equivalent circuit in the inset. Starting from the high frequency range (left side), a first semicircle is easily individuated which may be attributed to the response of the bulk silicon, not involved in the doping process; the second semicircle, at intermediate frequency, may be attributed to the response of the PSi layer. A linear trend about 45° sloped may be individuated in the last part of the spectrum, at the lowest frequencies, as well as a third semicircle, less defined with respect to the previous ones, attributable to diffusion of Er^+3^ ions which tend to accumulate near the pore surface.

Analogous discussion may be done on data obtained during high current doping (Figure 4b): in this case, the final part of the spectrum is better resolved and a further semicircle clearly appears. As shown in the inset of Figure 4b, a further circuital element was needed in the equivalent circuit to fit the related experimental data: a Warburg element W, corresponding to a CPE with *n* = 0.5 [18].

Different processes can be evocated to interpret this behavior, also considering the high values of cell potential which establish at high current. As reported in the literature [13-15], adsorption of Er ions, as well as redox processes involving molecular hydrogen and Er^+3^ itself, could occur in this condition. However, in the present work, no evidence of Er reductive peaks was found in the cyclic voltammetries carried out on pristine PSi layers in the same range of potentials (data not shown). Moreover, a jelly-like phase, constituted by Er ethanolate, has been observed following Er doping with similar parameters [14]. The presence of this jelly-like phase within the pores and the proportionality of the rate of the deposit formation to the current density have also been reported [13].

On the basis of these results, a possible interpretative model of the observed behavior can be proposed: the applied electric field induces a migration of the Er^3+^ ions present in the electrochemical solution towards the inner pores surface, so generating a distribution of charges inside the pores, as well as a charge transfer of the ions inside of the solid structure. These two processes originate two resistive/capacitive responses in the GEIS spectra (second and third circles in Figure 4a,b).

**Figure 5:**
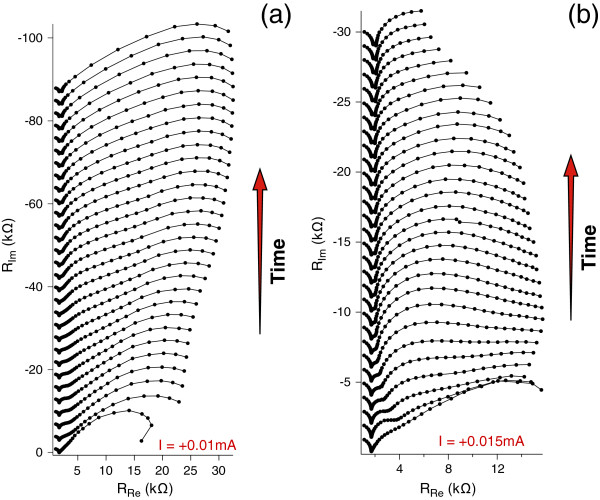
**Examples of GEIS results for low doping current intensities.** Evolution in time of Nyquist plots during the Er doping of two nominally identical PSi samples, 1.25 μm thick, carried out at low current intensities (*I* = +0.010 mA for **a** and *I* = +0.015 mA for **b**). For each section in the figure, the first measurement is the lowest curve. All GEIS cycles have been measured in sequence with an interval of about 4 s between a cycle and the next. Curves related to increasing times are shifted in the y-axis for reason of clarity, and an arrow indicating the direction of time is indicated.

**Figure 6:**
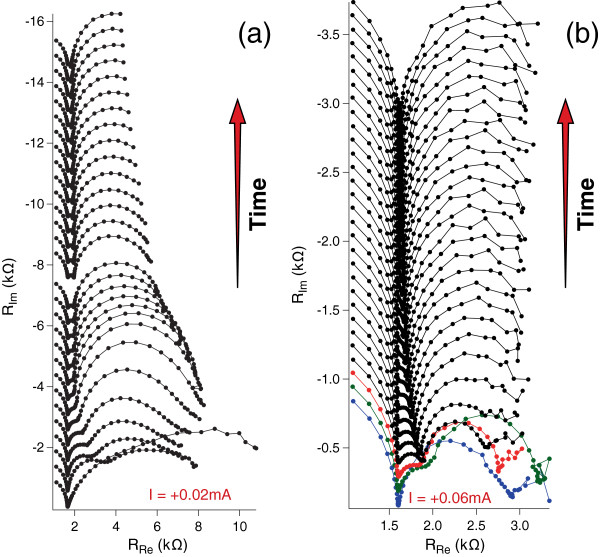
**Examples of GEIS results for high doping current intensities.** Evolution in time of Nyquist plots during the Er doping of two nominally identical PSi samples, 1.25 μm thick, carried out at high current intensities (*I* = +0.02 mA for **a** and *I* = +0.06 mA for **b**). For each section in the figure, the first measurement is the lowest curve. All GEIS cycles have been measured in sequence with an interval of about 4 s between a cycle and the next. Curves related to increasing times are shifted in the y-axis for reason of clarity, and an arrow indicating the direction of time is indicated. The colors are used for an easier reading of the evolution in the first stages of the process.

At high electric fields, the high ion flux in the liquid phase leads to a consistent Er^3+^ ion accumulation near the PSi surface up to a concentration high enough for the formation of the jelly-like layer, and in turn, a new interphase appears, originating the last semicircle in the spectra of Figure 4b.Finally, in order to derive information on the onset of the transients observed at different current doping, GEIS measurements were carried out applying different constant bias current densities, matching those used for the continuous doping of the samples of Figure 1. For each sample, a series of GEIS spectra were recorded, starting from the pristine PSi layer, so to follow the behavior observed for the continuous doping. In fact, since each GEIS cycle is identical to the others, we can assimilate the series of GEIS cycles to a sort of step-by-step doping.Figures 5 and 6 show some examples of the GEIS results, in terms of Nyquist diagram, performed on nominally identical samples using different constant bias currents (indicated in each figure). Each curve of a graph corresponds to a single GEIS cycle, and each point on a GEIS cycle is obtained at a single frequency. The first cycle in each series is at the bottom and the last at the top. Please note that the graphs of Figure 4 are the 4th and 3rd GEIS cycles of Figures 5a and 6b, respectively.The difference of the GEIS measurements results in Figures 5 and 6 is evident, and we associate the behavior shown in Figure 5 to the ST regime (lower currents) and the one in Figure 6 to the DT regime (higher currents).

According to the interpretation derived by the equivalent circuits, the first semicircle (from the left, higher frequencies) is attributed to the bulk Si. It does not evolve with time in each series of measurements, since bulk Si is not affected by the doping process. A variation of the diameters of the other semicircles is measured in time, at a variable extent, especially in data at highest current. The appearance/disappearance of the responses is connected with the time constants related to the different processes. From the fitting described earlier, values in the order of microseconds are obtained for the first RC element, so confirming a rapid process of charge adjustment in the bulk solid phase. Slower processes, represented by the other semicircles, are observed at lower current doping (time constants of order of 10^-1^ s), while an acceleration of them is observed at higher current (time constants in the order of ms). The presence of the DT can tentatively be associated to the large and rapid variation observed in the third semicircle in the higher current time evolution, not visible in the lower current measurements.

### EDS-SEM characterization

The GEIS and optical reflectivity measurements being not a direct Er concentration measurement, we resorted to energy dispersive spectroscopy by scanning electron microscopy (SEM-EDS) measurements to gain direct access to the presence of Er within the porous layer. The results are summarized in Table 1, where we report the evolution of the Er content with depth for two PSi samples doped using two doping current intensities different by one order of magnitude and with an identical total transferred charge. The depth at which the measurements were taken is indicated in the first column of the table. The area for each measurement was 8 μm^2^.

**Table 1:** EDS-SEM measurements of Er content

**Depth (μm)**	**Er (At%) at *I*** **= +0.5 mA**	**Er (At%) at *I*** **= +0.05 mA**
2	1.24	0.12
6	1.29	0.09
9	1.22	0.21
13	1.14	0.23
17	0.91	0.21
22	0.11	0.02

Er content (At%) for two PSi samples doped using two one order of magnitude-different doping currents. The samples were 20-μm thick, and the last point at 22-μm depth has been measured in the bulk Si region as reference for background signal.

The measured Er% for the sample doped using the lower current intensity is lower at all depths with respect to the other sample. Even if the Er% for this sample is below the quantitative threshold, the SEM-EDS measurements demonstrate that the total amount of Er deposited is significantly different for lower and higher current intensities despite the transferred charge and the PSi parameters being identical: lower currents lead to lower doping levels. It is not possible, at present, to correlate directly the Er distribution with our model and the GEIS measurements since the considered thicknesses are too different: 2.5 μm for GEIS and 22 μm for the EDS-SEM.

The SEM-EDS data give then further support to the already consistent interpretation of the optical and electrochemical measurements we described earlier, adding a direct measurement of the significant difference in the Er content for samples having as sole difference the doping current intensity. These results also strongly suggest that the doping current is a very good candidate to control and optimize the Er doping process of porous silicon.

## Conclusions

We demonstrate that the voltage transitory of constant-current Er doping of PSi samples is tightly related to the final doping level. From the shape of the transitory, it is possible to anticipate the effectiveness of the doping process: a qualitative correlation of the final Er content with the transitory shape has been evidenced. This work therefore shows that a good understanding and control of the initial steps of the Er doping process is a key to the optimization of the whole process itself. Although it is presently too early to determine which are the best Er-doping conditions for porous silicon, we demonstrate that the result of the doping process depends on the parameter settings and that the current intensity is a relevant doping factor.

## Abbreviations

DT: double transitory; EDS-SEM: energy dispersive spectroscopy by scanning electron microscopy; EIS: electrochemical impedance spectroscopy; GEIS: galvanostatic electrochemical impedance spectroscopy; PSi: porous silicon; ST: single transitory.

## Competing interests

The authors declare no competing interests.

## Authors’ contributions

The work presented here was carried out in collaboration between all authors. All authors have contributed to, seen, and approved the manuscript.
